# Evolution and Diversity of the *Microviridae* Viral Family through a Collection of 81 New Complete Genomes Assembled from Virome Reads

**DOI:** 10.1371/journal.pone.0040418

**Published:** 2012-07-11

**Authors:** Simon Roux, Mart Krupovic, Axel Poulet, Didier Debroas, François Enault

**Affiliations:** 1 Clermont Université, Université Blaise Pascal, Laboratoire “Microorganismes : Génome et Environnement”, Clermont-Ferrand, France; 2 Laboratoire “Microorganismes : Génome et Environnement”, Aubière, France; 3 Institut Pasteur, Unité Biologie Moléculaire du Gène chez les Extremophiles, Paris, France; Radboud University Nijmegen Medical Centre, NCMLS, Netherlands

## Abstract

Recent studies suggest that members of the *Microviridae* (a family of ssDNA bacteriophages) might play an important role in a broad spectrum of environments, as they were found in great number among the viral fraction from seawater and human gut samples. 24 completely sequenced *Microviridae* have been described so far, divided into three distinct groups named *Microvirus*, *Gokushovirinae* and *Alpavirinae*, this last group being only composed of prophages. In this study, we present the analysis of 81 new complete *Microviridae* genomes, assembled from viral metagenomes originating from various ecosystems. The phylogenetic analysis of the core genes highlights the existence of four groups, confirming the three sub-families described so far and exhibiting a new group, named *Pichovirinae*. The genomic organizations of these viruses are strikingly coherent with their phylogeny, the *Pichovirinae* being the only group of this family with a different organization of the three core genes. Analysis of the structure of the major capsid protein reveals the presence of mushroom-like insertions conserved within all the groups except for the microviruses. In addition, a peptidase gene was found in 10 *Microviridae* and its analysis indicates a horizontal gene transfer that occurred several times between these viruses and their bacterial hosts. This is the first report of such gene transfer in *Microviridae*. Finally, searches against viral metagenomes revealed the presence of highly similar sequences in a variety of biomes indicating that *Microviridae* probably have both an important role in these ecosystems and an ancient origin.

## Introduction

Viruses, particularly bacteriophages (viruses of bacteria), are the most numerous biological entities on Earth, retrieved from all sorts of biomes (human body, aquatic ecosystems, soil samples, etc.). Among these, phages with double-stranded DNA (dsDNA) genomes have been the most thoroughly studied [1]. A great deal of data is now available on their diversity [2], relationship with the hosts [3] and their evolution [4]. Such information is still lacking for single-stranded DNA (ssDNA) viruses, which were thought to be secondary actors in environmental viral communities. Yet, it has been recently discovered that these viruses are important members of the virosphere. Indeed, taking into account their modest genome sizes when compared to those of phages with dsDNA genomes, ssDNA viruses were identified in metagenomic datasets from a great variety of ecosystems [5]–[7]. Their ubiquity led virologists to focus specifically on these viruses [8], [9]. Among the ssDNA viruses, the *Microviridae* family is one of the most commonly retrieved.

*Microviridae* are small icosahedral viruses with circular single-stranded DNA genomes. This family has been thoroughly studied from numerous perspectives – from virion structure and assembly [10]–[13], to the mechanisms driving their evolution [14], and their stability in environmental conditions [15]. Based on structural and genomic differences, members of this family are further divided into two subgroups : microviruses (genus *Microvirus*) and gokushoviruses (subfamily *Gokushovirinae*) [16]. Very recently, a new tentative sub-family, the *Alpavirinae*, was found through bacterial genome analysis [17], and confirmed by metagenomic analysis [18]. The seven members of the genus *Microvirus* exclusively infect *Enterobacteria* and have been extensively studied through the archetype of this familly, the bacteriophage ΦX174 [10], [19]. *Gokushoviruses* are currently known to infect only obligate intra-cellular parasites, members of bacterial genera *Chlamydia*, *Bdellovibrio* and *Spiroplasma* [20]. Eleven completely sequenced *Gokushovirinae* genomes are currently available: 6 *Chlamydia* phages and 1 genome assembled from seawater viromes [9] are closely-related, whereas *Bdellovibrio* phage phiMH2K [20], *Spiroplasma* phage 4 [21], [22], Microvirus ΦCA82 [23], and another genome assembled from a seawater virome [9] are considerably more divergent. Description of *Alpavirinae* is restricted to prophages residing in the genomes of bacteria belonging to two genera of the phylum *Bacteroidetes*: *Prevotella* and *Bacteroides*. The latter study was the first to implicate the *Microviridae* in lysogenization of their hosts and also to associate this virus group with *Bacteroidetes* [17].

*Microviridae*-like sequences were found in large numbers in different ecosystems, ranging from microbialites [24] to a variety of aquatic environments, with their presence in the GOS dataset [17] and in viral metagenomes [5], [6]. Viromes from human stool [18], [25] and coral [7] samples were also found to contain *Microviridae*-like sequences. As the known members of the *Microviridae* family exhibit small genomes (3–7 kb), two complete *Microviridae* genomes could be assembled from the Sargasso Sea virome [9].

To gain insights into the diversity of the *Microviridae* viruses in the environment, we reanalyzed a set of previously published viromes by assembling the reads from each of these viromes and then searching the resultant contigs for the presence of complete genome sequences related to *Microviridae*. We were able to assemble 81 complete circular genomes related to members of the *Microviridae* from 95 public viromes. Phylogenetic and genomic organization analyses of these new viruses revealed a new *Microviridae* subgroup (the *Pichovirinae*), enriched the genome collection of *Gokushovirinae*, and, for the first time, confirmed the existence of extrachromosomal complete genomes from *Alpavirinae* virion particles. *Microviridae* core genes could be more thoroughly studied, especially the structure of the major capsid protein. Horizontal gene acquisition events are also documented for the first time in this viral family. Finally, as the viromes analyzed in the current study cover a wide range of ecosystems, the distribution of the new genomes inside the *Microviridae* tree provides a better understanding of both the diversity and the evolution of *Microviridae* family.

## Results

### Assembly of Complete Genomes

Even though sequences from *Microviridae* are found in a large number of viromes, assembly of complete *Microviridae* genomes was described in only one dataset, the Sargasso Sea virome [26]. Indeed, a consensus sequence of a Chp1-like *Microviridae* was first created by Angly *et al.*, [26] and two complete genomes affiliated to *Microviridae* were assembled in a recent analysis of the same dataset using up-to-date assembly software [9]. To further decipher the evolution and distribution of this family, we assembled all available public viromes (with a threshold of 98% identity on 35 bp) and screened them for the presence of complete *Microviridae*-like circular genomes. As a result, 81 contigs representing putative complete *Microviridae* genomes were obtained from 25 out of the 95 viromes tested (Table S1). Out of these 81 new genomes, 15 new *Microviridae* were assembled from freshwater virome reads, 2 from a marine sample, 2 from microbialites, 1 from coral, 2 from human lung and 59 from human gut samples. Obviously, the number of *Microviridae* genomes assembled from a virome depends on the length of the sequences (Table S1) but the assembly also depends on the relative abundance of a given virotype in the sampled ecosystem. The sizes of the different genomes generated were quite homogeneous (Table S2), with the smallest genome being 3,989 bp-long and the longest 6,723 bp. This size range is consistent with the genome sizes of known *Microviridae* (between 4.4 and 6.1 Kb).

In order to detect potential new prophages (viral genomes integrated into bacterial chromosomes), the newly assembled *Microviridae* sequences were used as queries in searches against the complete bacterial genomes from the NCBI and the Human Microbiome Project [27] databases. The five previously described complete prophages [17] were fully retrieved, alongside a new one, detected in the recently sequenced *Prevotella multiformis* strain, highly similar to other *Microviridae* prophages detected in *Prevotella.*

### Phylogeny and Genome Organization of the Microviridae Family

**Figure 1:**
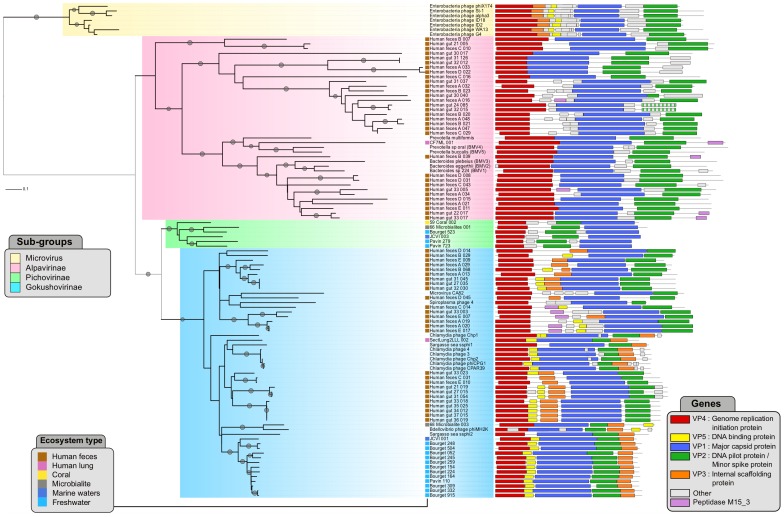
Phylogenetic tree drawn from the major capsid protein multiple alignment. Linearized genomes are represented for each virus. The open reading frames in each genome are color-coded following the nomenclature used for *Chlamydia* phage genomes (i.e.VP1 : major capsid protein, VP2 : DNA pilot protein, VP3 : internal scaffolding protein, VP4 : genome replication initiation protein, and VP5 : DNA binding protein). Striped-colored genes encode proteins possessing features characteristic of VP2 proteins, but displaying no significant sequence similarity, as assessed by BLAST. The four *Microviridae* subgroups are highlighted on the tree. Bootstrap scores greater than 80 are marked with gray dots.

In order to gain insight into the diversity of the *Microviridae* family and its genome evolution, a phylogenetic tree was computed from the major capsid protein (VP1) sequences and correlated with the corresponding genome maps (Fig. 1).

#### Phylogenetic analysis using the VP1 protein

Four well-supported clades (bootstrap values higher than 75) are formed on the VP1 phylogenetic tree: three of these correspond to the previously described taxonomic groups (*i.e.* genus *Microvirus,* and subfamilies *Gokushovirinae* and *Alpavirinae*), while the fourth one is exclusively composed of genomes generated in this study. The assembled viromes considerably expanded the available complete viral genome pool for *Gokushovirinae* and *Alpavirinae,* while not a single new virus was affiliated to the genus *Microvirus.* These four groups are further described below:

- The Enterobacteria phage group (the yellow group in Fig. 1) is exclusively composed of known members of the genus *Microvirus.* These phages are clearly separated from the rest of the *Microviridae*, consistent with the current ICTV taxonomy, where they form a distinct genus within the *Microviridae* family.
- The **Alpavirinae group** (the pink group in Fig. 1) includes the recently described *Alpavirinae* and 33 newly assembled *Microviridae* genomes. This group can be divided into three subgroups : two subgroups are exclusively composed of new genomes from the human gut flora. The first subgroup consists of 18 related genomes, while the second one encompasses three more divergent viruses Human_feces_B_007, Human_gut_21_005, and Human_feces_C_010. The third subgroup is composed of the 6 prophages, a new virus from a human lung sample and 11 viruses from the human gut samples. Notably, prophages from *Bacteroides* are separated from those inserted into the genomes of *Prevotella.* Consistently, the new *Prevotella* prophage (*Prevotella multiformis*) detected in this study is more similar to prophages from other *Prevotella* genomes. Interestingly, a new *Microviridae,* generated from free viral particles from a human lung sample, is interspersed on the tree among these *Prevotella* prophages. *Bacteroides* prophages form a monophyletic group with 11 new genomes sampled from human gut flora.
- The ***Gokushovirinae*** **group** (the blue group in Fig. 1) consists of all known *Gokushovirinae* and 42 newly assembled *Microviridae* genomes. These new viruses come from different ecosystems : 27 from the human gut flora (9 different individuals), 12 from freshwater lakes (11 from Lake Bourget and 1 from Lake Pavin), 1 from marine environment (JCVI_001, sampled from Chesapeake Bay), 1 from human lung (SectLung2LLL_002) and 1 from microbialites (68_Microbialites_003). This group can be internally divided : *Gokushovirinae* assembled from aquatic samples are most closely related to *Bdellovibrio* phage ΦMH2K, whereas sequences assembled from human gut samples are divided into two subgroups, one around *Spiroplasma* phage 4 and Microvirus ΦCA82, and another group close to *Chlamydia* phages.
- The **new group** (the green group in Fig. 1) is composed exclusively of new viruses assembled from metagenomic sequences. This group contains 3 genomes from two different freshwater lakes, 1 from marine water, 1 virus associated with coral microbiota and 1 from microbialites. As this group is separated from the already defined groups, we propose to name it ***Pichovirinae*** (*Picho* : small in Occitan).

#### Genome analysis of Microviridae

All 81 new *Microviridae* genomes were composed of 3 to 9 predicted genes. These gene numbers are consistent with the known reference genomes, and were similar for each subgroup. The *Microviridae* core genes (encoding the major capsid protein VP1, minor spike or pilot protein VP2 and replication initiation protein VP4) are detected in all *Microviridae* genomes but two (Fig. 1). The average genome size of the four different sub-families was found to be significantly different (Fig. S1, one-way ANOVA, p-value 2.2e-16). Notably, *Microvirus* and *Alpavirinae* genomes are longer than those of *Pichovirinae* and *Gokushovirinae.* Furthermore, the genomic organization of each of the 4 sub-families is specific. Indeed, the genome organizations are conserved within the 4 sub-families but different between the sub-families (Fig. 1).

Genomes of *Alpavirinae* display a reduced content of *Microviridae* conserved genes, with only three genes (for proteins VP1, VP2 and VP4) being significantly similar to those of *Microviridae* from other genera/subfamilies. Two new genomes (Human_gut_24_085 and Human_gut_32_015), sampled from two different individuals, lack an ORF significantly similar to VP2, but instead possess a similarly-sized ORF at a position equivalent to that occupied by VP2 in all other members of the *Alpavirinae* (Striped-colored genes on Fig. 1). These ORFs are likely to be highly divergent VP2-coding genes. Consistently, the putative products of both ORFs display features characteristic of all VP2-like proteins. Namely, both gene products possess predicted N-terminal transmembrane domains and coiled-coil regions. Although no other microviral genes could be detected within the *Alpavirinae* prophages and related assembled genomes using sequence-based searches, it has been previously suggested that VP3-like scaffolding proteins might be encoded transcriptionally downstream from the VP1-encoding genes [17]. Indeed, most of the *Microviridae* from the *Alpavirinae* group possess unassigned ORFs that might encode an equivalent of gokushoviral protein VP3.

Genomes of *Gokushovirinae* share the same gene content (presence of ORFs significantly similar to VP1, VP2, VP3, VP4 and VP5), with the exception of *Spiroplasma* phage 4, Microvirus ΦCA82, Sargasso sea phage ssΦ2 and 12 new genomes from human gut, for which VP3 and/or VP5-like genes could not be identified using standard sequence analysis methods. However, upon a closer examination using a sensitive profile-profile comparison algorithm implemented in FFAS03 [28], an ORF potentially encoding a homologue of VP3 has been identified in 4 of these genomes from human gut (Human_Gut_33_003, Human_Feces_A_019, Human_Feces_A_020, Human_Feces_E_017; hit to *Chlamydia* phage Chp2 scaffold protein VP3 superfamily, pfam id : PF09675; FFAS03 mean score: −29.2). An internal separation of the *Gokushovirinae* assembled in this study into two subgroups can be deduced from the gene order conservation within these subgroups, consistently with the phylogenetic information. A first subgroup is found near *Bdellovibrio* phage ΦMH2K, and encompass only genomes assembled from aquatic environments (Lake Bourget, Lake Pavin, and JCVI_001, sampled from Chesapeake bay). This subgroup displays a specific gene order : VP4-VP5-VP1-VP2-VP3 (Fig. 1). Genomes assembled from human gut present a different gene order (VP4-VP5-VP3-VP1-VP2), and do not form a monophyletic group within the *Gokushovirinae.* Yet, the low bootstraps scores point towards the possibility that the internal branching within this group might change once more gokushoviral sequences become available. Finally, two exceptions have to be noted : first, a sequence assembled from Human Lung sample (SectLung2LLL_002) is found near the known *Chlamydia* phages, and presents a gene order similar to the aquatic *Gokushovirinae* assembled in this study (Fig. 1, VP4-VP5-VP1-VP2-VP3); second a genome assembled from Microbialites (68_Microbialites_003) displays the same gene order as *Bdellovibrio* phage ΦMH2K, and is related to this phage in the tree with a significant bootstrap support.

The gene composition of *Pichovirinae* genomes is similar to that of *Alpavirinae*: significant sequence similarity with known references are only detected for the three major genes (for VP1, VP2 and VP4). Nevertheless, *Pichovirinae* genomes are the only ones within the *Microviridae* family where the gene order of the core genes is altered: whereas all *Microviridae* present a VP4 - VP1 - VP2 organization, the gene order in all *Pichovirinae* is VP4 - VP2 - VP1 (Fig. 1).

### Detailed Analysis of the Conserved Microviridae Proteins

#### Major capsid protein (VP1)

**Figure 2:**
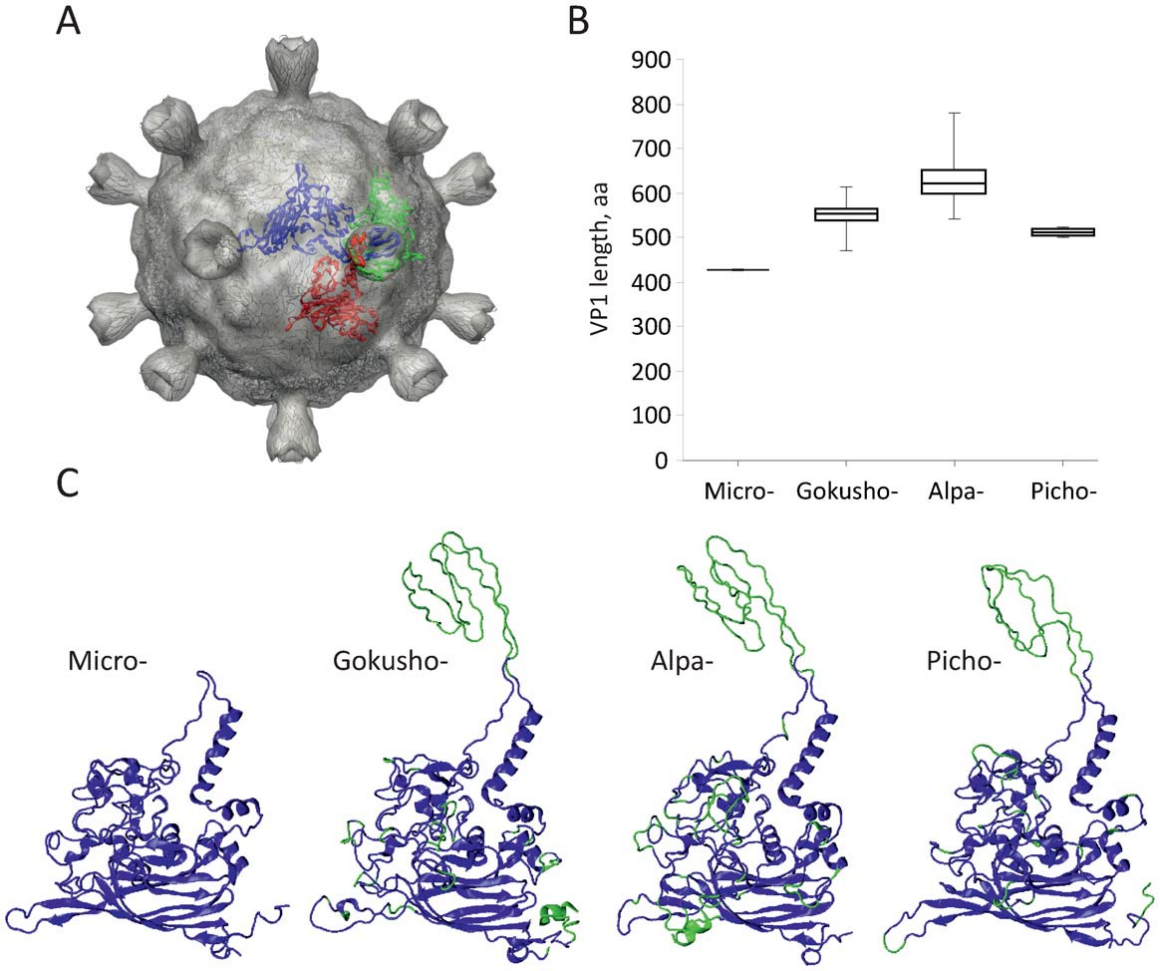
Major capsid protein (MCP) variation within the *Microviridae*. (A) Three-dimensional model of the SpV4 virion (PDB ID:1KVP). Three capsomers donating long insertion loops to form the ‘mushroom-like’ protrusions at the three-fold axes of symmetry of the icosahedral capsid are highlighted in blue, green, and red. (B) A boxplot illustrating the variation of MCP sizes between the four subgroups of the *Microviridae*. (C) Three-dimensional models of the MCPs from viruses representing the four subgroups of the *Microviridae*: *Microvirus* (ΦX174 protein F; PDB ID:1CD3), *Gokushovirinae* (SpV4 VP1, PDB ID:1KVP), *Alpavirinae* (*Prevotella bucalis* prophage BMV5 protein VP1; GI:282877220), *Pichovirinae* (Pavin_279 protein VP1). The insertions within the VP1 proteins of gokusho-, alpa- and pichoviruses relative to the F protein of ΦX174 are highlighted in green.

Virions of microviruses and gokushoviruses display distinct structural features and molecular composition; although both possess icosahedral capsids composed of 60 copies of the major capsid protein, MCP (F and VP1, respectively), only those of microviruses are decorated with pentameric major spike protein complexes positioned at each of the 12 five-fold vertices [11]. Electron cryo-microscopy (cryo-EM) study of the SpV4 virions revealed that gokushoviruses instead possess 55 Å-long ‘mushroom-like’ protrusions located at the 3-fold symmetry axes of their capsids [21]. These protrusions are formed by insertion loops coming from three subunits of the VP1 protein (Fig. 2A), and were suggested to participate in receptor recognition and binding on the host cell surface. The protrusion-forming insertion is not present in the MCPs of ΦX174-like microviruses and is largely accountable for the size differences between the MCPs of microviruses and gokushoviruses (Fig. 2B).

Comparison of the MCP sequences from the four subgroups of the *Microviridae* revealed that the average identity at the protein level varies from 40 to 80% within a group, and from 20 to 40% between the groups, with an exception of the *Enterobacteria*-infecting phages, which generally present no significant sequence identity with MCPs from other clades of the *Microviridae* (Fig. S2). Analysis of the VP1 size variation including the newly discovered members of the *Microviridae* revealed that the average MCP size in the four different sub-families is significantly different (one-way ANOVA, p-value 1e-43). ΦX174-like microviruses possess smaller MCPs (average length 427 aa), while those of gokusho-, alpa- and pichoviruses are significantly larger (Fig. 2B). Notably, among the latter three groups, pichoviruses possess the smallest MCPs (average length 512 aa), while the MCPs of alpaviruses are the largest (average length 630 aa) and also the most variable in terms of size (ranging from 541 to 780 aa). Multiple sequence alignment of VP1 homologues revealed that the MCP size difference is a result of variation in the number and size of insertions in VP1-like proteins (Fig. S3).

To gain insights into possible architecture of viruses from the newly identified groups *Alpavirinae* and *Pichovirinae* and to understand the effect that the insertions within their MCPs might have on virion architecture, we constructed three-dimensional VP1 models for representative viruses. These were compared to the available X-ray structure of ΦX174 protein F (PDB ID:2BPA; [11]) and the cryo-EM-based model of SpV4 protein VP1 (PDB ID:1KVP; [21]). Structural modeling and model quality assessment are described in Materials and Methods. Comparison of the structural models (Fig. 2C) revealed that VP1 homologues from viruses belonging to all four groups of the *Microviridae* possess a conserved eight-stranded β-barrel core (also known as viral jelly-roll; [29]) and all, but ΦX174-like microviruses, possess an extended loop that forms a mushroom-like protrusion in SpV4. Consequently, it is likely that virions of gokusho-, alpa- and pichoviruses, unlike those of microviruses, possess characteristic receptor-binding spikes at the three-fold axes of the icosahedral capsids (Fig. 2A).

Further analysis has revealed that the size of the putative receptor-binding spike-forming insertions differs between different subgroups of *Microviridae* (Fig. S4A): the shortest are found in pichoviruses (average length 60 aa), while those of alpaviruses are the longest (average length 110 aa). The insertion length also varies considerably within *Gokushovirinae* (from 53 to 114 aa) and *Alpavirinae* (from 45 to 209 aa). Interestingly, this variation appears to be ecosystem- rather than virus subgroup-dependent. The insertion length variation was much less pronounced for VP1 proteins of viruses residing in aquatic environments (including both gokushoviruses and pichoviruses) than it was for viruses from human samples (gokushoviruses and alpaviruses) (Fig. S4B). The same tendency was also true for the full length MCPs, with VP1s from human samples being larger (average length 604 aa) than those of viruses thriving in aquatic environments (average length 533 aa). It therefore appears that evolution towards acquisition of insertions within the MCPs of viruses isolated from human samples might be driven by the need to cope with additional factors (e.g., immune system, low pH of the human gastrointestinal tract, etc.) that are not present in aquatic environments.

Distinct members of the *Alpavirinae* (a group exclusively associated with human samples; Fig. 1) possess insertions at different locations within their VP1 proteins, suggesting that VP1 proteins within this *Microviridae* subgroup are indeed evolving rapidly. Such species-specific insertions were found to be up to 231 aa-long (Human_feces_B_007). In order to verify whether such extensive insertions would not interfere with normal virion formation, we fitted our three-dimensional model of the alpaviral VP1 into the pseudoatomic model of SpV4 (PDB ID:1KVP) and mapped the location of all the insertions exceeding 15 aa. We identified 6 MCP hot-spots where large insertions were tolerated in alpaviruses (Fig. S5). Notably, all of these insertions occurred in the loop regions of the MCP facing outwards from the virion surface and are therefore expected not to affect virion assembly. Interestingly, the 231 aa-long insertion in the MCP of Human_feces_B_007 is predicted to be rich in β-strands and is likely to fold into an independent domain. Peculiarly, the major spike protein G of ΦX174-like microviruses, which forms protrusions at the virion five-folds of these viruses is also rich in β-strands. Unfortunately, the sequence of this insertion in the MCP of Human_feces_B_007 does not share significant similarity with proteins in extant databases and its provenance therefore remains obscure.

#### Replication protein (VP4)

The replication protein is highly variable in length, as some microphages possess long replication genes (namely *Alpavirinae* assembled from prophages and the associated virions, but also *Chlamydia* phage Chp1, and Sargasso sea phage ssphi1). Nevertheless, the three conserved motifs of superfamily I rolling cycle replication proteins are all conserved (Fig. S6), suggesting that these proteins are likely to be functional. High levels of sequence identity are detected within all *Enterobacteria* phages sequences, as well as within *Pichovirinae and Gokushovirinae* (Fig. S7). Conversely, replication proteins from *Alpavirinae* are considerably less conserved within the group. Globally, the similarity between VP4 sequences for any given pair of viruses is lower than the one for the VP1 sequences from the same pair of viruses.

#### DNA pilot protein (VP2)

The last gene retrieved in all *Microviridae* genomes to date codes for the pilot protein (VP2 in *Gokushovirinae,* Minor spike protein H in *Enterobacteria* phages). The ΦX174 protein H is a multifunctional structural protein (12 copies per virion) required for piloting the viral DNA into the host cell interior during the entry process, and *de novo* synthesis of protein H is required for efficient production of other viral proteins [30]–[32]. However, the full functional potential of this protein remains to be elucidated. At the first glance, VP2 appears to be more divergent than the MCP or replication protein: significant sequence similarity is only detected within sequences of the same subgroup (*Gokushovirinae, Enterobacteria phages*, *Alpavirinae* and *Pichovirinae,* Fig. S8). Strikingly however, similarity between VP2 proteins from more closely related viruses often equals or even exceed the similarity observed between their major capsid or replication proteins. This is, for example, the case for *Chlamydia* phages (with the exception of the highly divergent *Chlamydia* phage 1; Fig. S8), and *Enterobacteria*-infecting *phages*. This perplexing host-dependent pattern of VP2 conservation raises the possibility that the evolution and function(s) of this protein might be tightly linked to the identity of the host.

### Horizontal Acquisition of New Genes

It has been previously suggested that genes encoding novel functions in microviral genomes emerge from pre-existing genomic regions through accumulation of point mutations [19], [33]. This conclusion has been supported by the lack of identifiable cases of horizontal acquisition of new genes by the *Microviridae*. Analysis of the complete microviral genomes assembled in this study has unexpectedly revealed 11 genes from human-associated *Microviridae* (5 *Gokushovirinae* and 6 *Alpavirinae -* 10 from human gut and 1 from human lung; Fig. 1) encoding a putative peptidase of the M15_3 family (Pfam Id : PF08291). M15 family peptidases are widespread in bacteria and are involved in cell wall biosynthesis and metabolism; they catalyze hydrolytic cleavage of the amide bond within peptide bridges that cross-link glycan strands of the bacterial cell wall [34].

**Figure 3:**
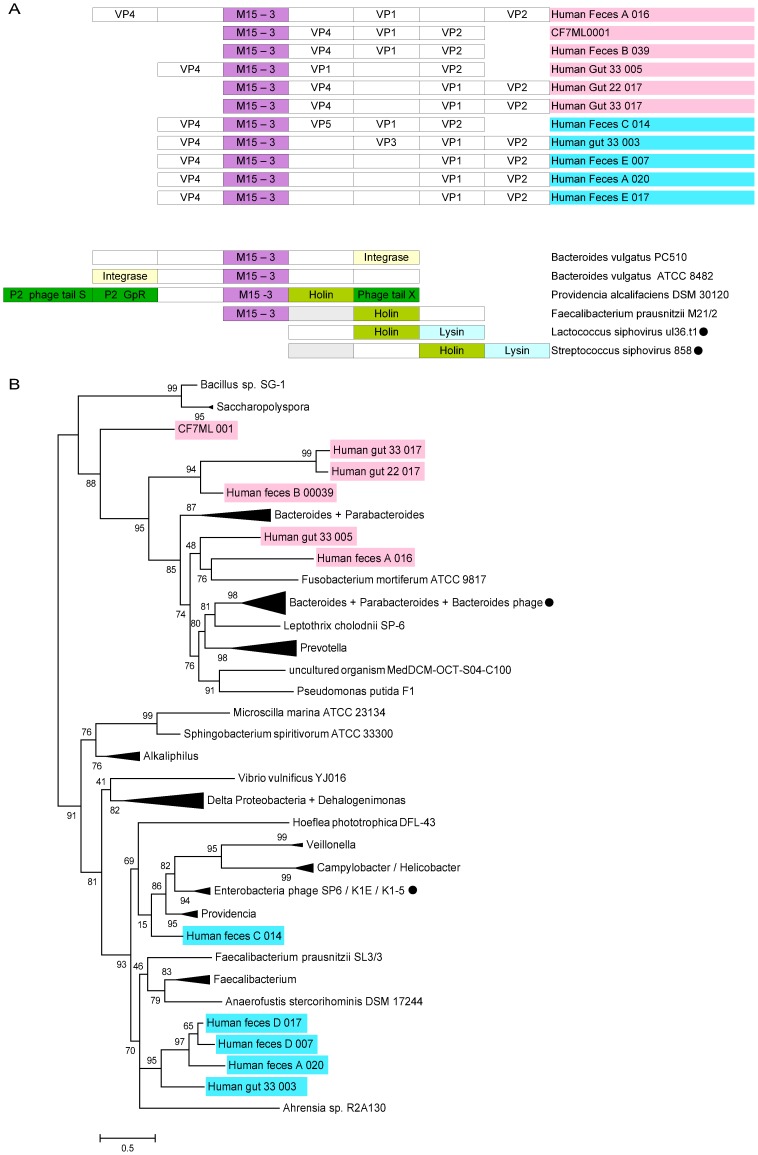
Genomic context and phylogenetic analysis of the Peptidase M15 *Microviridae* sequences. **(**A) Organization of the Peptidase M15_3 region in the 11 newly assembled *Microviridae* genomes. The regions encompassing homologous peptidase genes in three bacterial and two phages (noted with a black circle) genomes are also shown. *P2 GpR* stands for the P2 phage tail completion protein. (B) Maximum-likelihood tree computed from the multiple alignment of peptidase M15 sequences of the *Microvidae* and their closest homologues in viral and bacterial genomes. Bootstrap support values are indicated on each node. A fully expanded view of this tree is available as Fig. S9.

The closest homologues detected by BLAST for these 11 genes are from bacterial genomes, except for Human_feces_A_016, for which the closest homologue is found in a tailed dsDNA phage genome (Table S3). Half of the *Alpavirinae* peptidase genes are affiliated to *Bacteroidetes* and a *Bacteroidetes* phage, the three others are associated with *Burkhorderiales* (*Leptothrix* and *Collimonas*). *Gokushovirinae* peptidases are affiliated to *Firmicutes* : *Faecalibacterium prausnitzii* (4 of 5), and *Gamma-proteobacteria* (*Providencia alcalifaciens*). These closest homologues of the microviral proteins are found next to phage-like genes in several bacterial genomes (Fig. 3A). For example, phage-like integrase genes are proximal to the M15 peptidase genes in *Bacteroides vulgatus ATCC* 8482 and *Bacteroides vulgatus PC510* genomes, indicating a likely phage origin for these genomic regions. Consistently, the peptidase gene from *Providencia alcalifaciens DSM30123* genome is retrieved within a complete prophage region, and next to a putative holin gene. The peptidase gene from *Faecalibacterium prausnitzii* M21/2 is present within a three-gene cassette, with all three genes having homologues in bacteriophages (Fig. 3A). Notably, besides the M15 peptidase, the cassette includes a putative holin gene (hit to *Lactococcus* phage ul36.t1, ABD63797; 47% identity, E = 1e-20) and a gene of unknown function, with a homologue present as part of the lysis gene cluster in *Streptococcus* phage 858 (Fig. 3A). All this indicates that peptidase genes are likely to be frequently exchanged between viruses and their hosts, probably through prophage integration. Interestingly, 5 of the 11 peptidase genes detected in *Microviridae* genomes are adjacent to an “unknown” predicted ORF, which displays no sequence similarity to proteins in the extant databases. Peculiarly, this unknown gene (153 codons) in the Human_gut_33_003 genome is encoded on the complementary strand. Such orientation is highly unusual; to our knowledge, no other cases of complementary strand genes have been reported in *Microviridae*.

In order to shed light on the evolutionary event(s) leading to the acquisition of M15 peptidase genes by *Microviridae* phages, a phylogenetic tree was computed from a multiple alignment including the *Microviridae* peptidases and their closest homologues in both bacterial and viral genomes (Fig. 3B, Fig. S9). The topology of the peptidase tree is consistent with the VP1-derived tree of *Microviridae*, with a clear separation between the peptidase genes from the *Alpavirinae* (highlighted in pink) and the five peptidase genes from *Gokushovirinae* (in blue). Within the *Alpavirinae*, the peptidase tree topology can be associated with the location of the peptidase gene integration within these viral genomes. Peptidases genes are inserted in two different positions in *Alpavirinae* genomes : between VP2 and VP4 (CF7ML001, Human_feces_B_039, Human_gut_22_017 and Human_gut_33_017), and between VP4 and VP1 (Human_feces_A_016 and Human_gut_33_005) (Fig. 1). Consistently, these two groups are retrieved on the peptidase tree: Human_feces_A_016 and Human_gut_33_005 are found near the *Fusobacterium* gene within the *Bacteroidetes* group, whereas the other *Alpavirinae* peptidases are retrieved at the base of the *Bacteroidetes* group. Within the *Gokushovirinae*, the Human_feces_C_014 peptidase is separated from the rest of gokushoviral peptidases, similarly to the topology of the VP1 tree (Fig. 3B, Fig. 1). In the peptidase tree, the closest neighbors of the *Gokushovirinae* peptidases are from *Firmicutes* (*Faecalibacterium)* and *Proteobacteria* (*Ahrensia*, *Providencia*). The most likely explanation for such clustering is that peptidase genes were horizontally acquired by several members of the *Microviridae* on multiple occasions. The presence of dsDNA phage peptidases near the *Microviridae* sequences on the tree suggests that ds and ssDNA phages might be engaged in gene exchange, either directly during a co-infection or via infection of a prophage-bearing host cell.

Finally, the possibility of horizontal gene transfer between *Microviridae* genomes was investigated in light of this acquisition of a peptidase gene by several human gut *Microviridae.* For that, we performed a phylogenetic analysis of the two most conserved *Microviridae* proteins, VP1 and VP4, for each of the four subgroups (*Microvirus, Alpavirinae, Gokushovirinae* and *Pichovirinae*). No signs of recent gene transfer event could be detected, confirming the hypothesis that gene transfer between *Microviridae* are rare, even within the temperate members of the group [14], [17], [19].

### Microphages Diversity in Environment

#### Biogeographic pattern and Microviridae dispersal

The wide distribution of samples from which complete *Microviridae* genomes could be assembled (Table S1) makes it possible to analyze the repartition of the different *Microviridae* subgroups among the different geographic sites sampled, and the different types of environments studied. Remarkably, *Microviridae* genomes could be assembled from all but hypersaline and hyperthermophilic types of samples. Very similar genomes are retrieved from geographically remote sampling sites, both in aquatic medium (for examples JCVI_001 from North America is closely related to Bourget_248 and Bourget_504, from France) and in human microbiome : genomes noted as “Human feces”, sampled in South Korea [18], are not very different from the “Human gut” genomes, sampled in North-America [25]. This wide distribution and absence of biogeographic pattern is likely to reflect an ancient origin for *Microviridae*, which would have colonized a wide range of habitats, from human microbiome to seawater, freshwater, and sedimentary structures like Microbialites.

Nevertheless, assembling complete *Microviridae* genomes from random environmental sequences requires a large number of reads, especially for viromes composed of reads not exceeding 100 bp. Thus, only 3 complete *Microviridae* genomes have been generated from the three viromes with reads of ∼100 bp (from a total of 41 such viromes in the dataset, Table S1). To gain further insights into the diversity and patterns of distribution of *Microviridae,* a database of major capsid protein (VP1) sequences was built encompassing all published and newly assembled *Microviridae*. These sequences were used to search for VP1 homologues in the unassembled virome reads.

#### Viral metagenome sequences similar to VP1

**Figure 4:**
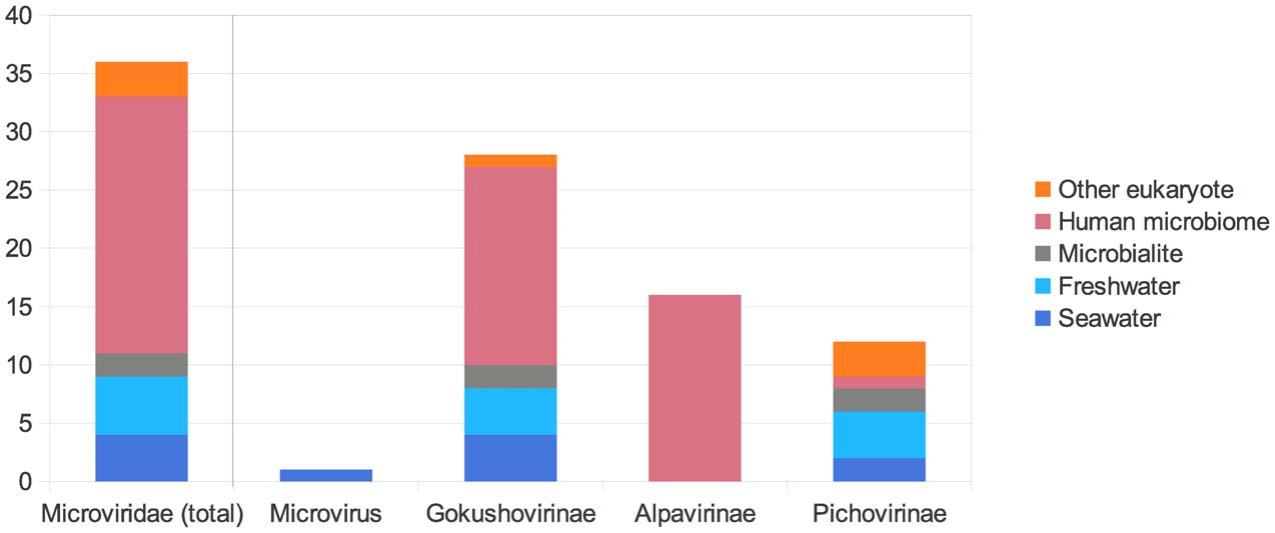
Abundance and distribution of VP1-like sequences in the environment. The number of viromes and the origin of the samples used for virome preparation are indicated. VP1 sequences were affiliated by best BLAST hit against a database including VP1 sequences from both the previously published *Microviridae* genomes and the complete genomes assembled in this study.

A total of 498 sequences were found to be significantly similar (BLASTx bit score greater than 50) to the VP1 protein of at least one of the *Microviridae* complete genomes. These 498 metagenomic sequences span 36 of the 95 viromes, from 5 different types of ecosystem (human microbiome, other eukaryote, seawater, freshwater, microbialites). *Microviridae* remains undetected in hypersaline and hyperthermophilic environments (Fig. 4). In order to analyze the dispersal of each *Microviridae* subgroup, the presence of each subgroup was checked in the 36 viromes containing *Microviridae* sequences. The *Gokushovirinae* subgroup is the most widespread among *Microviridae* (28 viromes out of 36), and is found in all *Microviridae*-containing biomes (Fig. 4). *Pichovirinae* are less frequently detected (12 viromes), but are also retrieved from different types of biomes. On the contrary, *Alpavirinae* are exclusively detected in human sample viromes (16 samples). Finally, only one sequence affiliated to the genus *Microvirus* was detected in a seawater virome. Yet, the low BLAST bit score (50.1) and the fact that this is the only microvirus-like sequence retrieved indicates that microviruses are likely to be extremely rare in such environments.

## Discussion

Microphages are progressively retrieved from a broad range of environmental samples from various locations. In this study, the focus on this family through a search in viral metagenomes made it possible to describe a new subgroup of *Microviridae* and considerably expanded the existing knowledge on genome evolution, diversity and environmental distribution of this viral family. Using already published viromes that have never been analyzed for the presence of complete *Microviridae* genomes, this study more than tripled the number of complete genomes available for this family.

### Technical Issues and Potential Bias of the de novo Metagenomic Assembly of Viral Genomes

Several points have to be discussed regarding both bioinformatical and biological issues in order to better understand the results obtained in this study. First, sequences reconstructed here from metagenomic data represent consensus sequences of individual microviral populations and thus, DNA sequence variations within each population are masked. Nevertheless, the assembly criteria used here (98% similarity on 35 bp) are considered stringent enough to gather only sequences from the same viral species [26]. Such criteria both limit sequence variations within each assembled genome and mask pyrosequencing errors. Second, even if *Microviridae* seems more abundant and frequently retrieved in particular ecosystem types (as in the human gut), all biomes were not evenly sampled. Indeed, the published viromes used in this study were generated independently and with different sequencing technologies. For instance, viromes from the human gut contain approximately 2 times more base pairs than viromes from sea water, and 6 times more than those from hypersaline ponds. Third, the methodology used to generate a virome greatly influences the type of viruses which will be retrieved. Viromes prepared through LASL (Linker Amplified Shotgun Library) are not supposed to contain any ssDNA genomes as this technique only recover dsDNA fragments. Not surprisingly, no *Microviridae* were detected in the two viromes prepared through LASL in this study, both sampled from hyperthermophilic environments. Last, the quantitative importance of ssDNA viruses in general, and of *Microviridae* more specifically, remains an open question. The abundance of *Microviridae* was first considered to be higher than predicted when the affiliation of virome reads was normalized by the mean genome length, *i.e.* when viruses were compared in terms of “number of viral particles in the original samples” [5]. Nevertheless, this quantitative importance was balanced by a potential bias of the whole-genome amplification methodology, which would preferentially amplify small circular DNA templates [35]. Although the real relative abundance of the *Microviridae* is still unknown, the fact remains that a considerable part of reads in numerous viromes could be affiliated to *Microviridae*, leading to the assembly of 81 complete *Microviridae* genomes.

### Microviridae is a Coherent Family of ssDNA Phages

The new genomes assembled in this study confirmed that *Microviridae* is a consistent and homogeneous viral family. All but two genomes contained ORFs significantly similar to the three core genes of *Microviridae* (the major capsid protein VP1, the replication protein VP4, and the DNA pilot protein VP2). Moreover, the division of the *Microviridae* family into sub-families, which has been previously presented [20], was both confirmed and complemented by this study. The microphage diversity deduced from the analysis of the environmental samples confirmed the distinction between *Enterobacteria-*infecting phages (genus *Microvirus*), and the other known *Microviridae.* Members of the genus *Microvirus* are considered as *Microviridae* archetypes, and are the most studied *Microviridae* so far, yet none of the genomes assembled in this study is associated with this genus. This apparent paradox might be due to culture bias, as *Microviruses* are cultivated on *E. coli* strains, the most widely used and studied prokaryotic model organism. The present study indicates that this genus is rare among the biosphere, and likely constitute a very specific type of *Microviridae* especially in terms of gene content and capsid structure. The other *Microviridae* appear to be internally divided into three main subgroups, namely *Gokushovirinae*, *Alpavirinae*, and a new subgroup, which we propose to name *Pichovirinae*. This new clade appear to be more related to *Gokushovirinae* than to *Enterobacteria* phages, and could represent an intermediate group, like *Alpavirinae*, which could fill the gap between the two currently recognized sub-families. Moreover, this new group has a unique genome organization, as VP2 is located between VP1 and VP4 for all the genomes in this group. Based on this synteny break, and the fact that *Pichovirinae*-like genomes have been assembled from very different samples (namely seawater, freshwater, microbialites, and coral samples), this clade has probably diverged from the common *Microviridae* ancestor a long time ago. Consistently, significant sequence similarity between pichoviruses and the other *Microviridae* is confined to the major capsid protein VP1 and the replication protein VP4, while the pilot proteins (VP2) displays only limited sequence similarity to corresponding proteins from the *Alpavirinae* prophages.

This taxonomic structure of the *Microviridae* family confirms that the microphage diversity was under-estimated. The detection of sequences homologous to *Pichovirinae* and *Gokushovirinae* in very different environments (Fig. 4) suggests that the viruses giving rise to these clades have probably diverged from their common ancestor in a distant past. The latter possibility is supported by the differential gene order conservation in the *Pichovirinae* on one side (VP1-VP4-VP2) and the remaining *Microviridae* subgroups on the other (VP1-VP2-VP4). The finding that microphages belonging to different subgroups occupy a variety of different ecological niches suggests that the association of microphages with bacteria is ancient, possibly predating the divergence of this cellular domain into the contemporary lineages.

### Major Capsid Protein Structure and Evolution

Based on similarity in virion architecture, it has been previously suggested that members of the *Microviridae* might share a common origin with eukaryotic viruses from the families *Circoviridae*, *Geminiviridae* and *Parvoviridae* [10]. Indeed, all these ssDNA viruses utilize eight-stranded β-barrel capsid proteins to build their icosahedral (T = 1) virions [36], [37]. However, while the capsid proteins of *Microviridae* and parvoviruses possess long insertion loops connecting the β-strands (although at different locations; [36]), those of geminiviruses and circoviruses are much more compact [37], [38]. Consequently, if the structural relationship indeed testifies for the common origin of these viruses, the evolution of *Microviridae* virion structure most likely proceeded through acquisition of insertion loops within the eight-stranded β-barrel core. As revealed through comparative analysis and structural modeling of the MCPs presented in this study, such dynamics within the loop regions of microviral MCPs appears to be an ongoing process, possibly assisting host-range expansion and adaptation to new environments in this viral family. This is especially obvious for microphages associated with human microbiota (all alpaviruses and certain gokushoviruses) that on average possess larger and more numerous insertions within their MCPs (Fig. 1, Fig. S4B). Paradoxically, although ΦX174-like phages are also known to infect hosts isolated from human samples [39], their MCPs are the most compact among the *Microviridae*. Interestingly, the putative receptor-binding spikes present at the three-fold symmetry axes of gokushovirus capsids ([21]; Fig. 2A) are also likely to decorate the virions of alpaviruses [17] and pichoviruses (Fig. 2C). The presence of this protrusion in all gokusho-, alpa- and pichoviruses, suggests that this feature is ancestral to the spikes present at the five-fold vertices of ΦX174-like microvirus capsids.

The number and the size of insertions within the microviral MCPs were similar in both prophages and free-living viruses, suggesting that these sequence modifications do not preclude the formation of viable virions. This specific evolutionary pattern of human microbiome *Microviridae* MCPs is reminiscent to co-evolution consequences described for cultivated phages. As described in the experiment of Paterson *et al.* [40], the basis of co-evolution is the absence of “non-adapted” host for the phage. This is consistent with the restriction of these viruses to human gut flora, where the highest bacterial densities for a microbial habitat were found [41]. Thus, human gut *Microviridae* are likely to be exposed to a higher host-phage encounter frequency compared to other *Microviridae*, thereby increasing the evolution rate of their MCP.

### Horizontal Gene Acquisition of a Possible Endolysin Gene

Until now, genes encoding novel functions in microviral genomes were thought to emerge from pre-existing genomic regions through accumulation of point mutations [19], [33]. However, the discovery in the *Microviridae* genomes of peptidase coding genes that were clearly acquired by horizontal gene transfer (HGT), and more likely through at least two independent transfer events, shows that *Microviridae* are able to integrate genes of interest from external sources into their genomes, even if such transfers are rare. The different uncharacterized ORFs detected in the new *Microviridae* genomes are then of great interest, since they could represent other horizontally acquired genes. On the contrary, no direct gene transfers between two *Microviridae* genomes could be detected in our dataset. Notably, phylogenetic analysis of the 47 closely-related *Escherichia coli*-infecting microviruses illuminated a few cases of HGT between these viruses that probably occurred by homologous recombination [14]. It is possible that HGT in *Microviridae* is limited by the genetic distance between the donor and the recipient virus species. Consequently, larger datasets of closely related virus genomes might be needed to better understand the prevalence of homologous recombination-driven HGT events in *Microviridae*.

ΦX174 is the only microvirus for which the mechanism of host cell lysis has been elucidated. Unlike dsDNA phages that typically encode a holin-endolysin system, where holin perforates the cytoplasmic membrane and endolysin digests the peptidoglycan, microviruses depend on a single-gene lysis system [42]. It has been shown that protein E of ΦX174 induces lysis by inhibiting cell wall biosynthesis [43]. It was therefore surprising to discover that gene for M15_3 peptidase identified in several gokushoviral and alpaviral genomes is associated with canonical lysis genes (for holin and endolysin) in dsDNA (pro)phages (Fig. 3A), suggesting that phage-encoded M15 peptidases might play a role in cell lysis during virus progeny release. Indeed, endopeptidase PLY500 (family VanY; PF02557), which is structurally related to M15 family proteases (families M15_3 and VanY belong to the same clan – Peptidase_MD; CL0170), acts as an endolysin at the end of the infection cycle of *Listeria* phage A500 [44]. We therefore suggest that *Microviridae* M15 peptidase might also be involved in dissolution of the host cell wall at the end of the phage life cycle.

### Microviridae Life Cycle and Putative Bacterial Hosts

From the current knowledge on *Microviridae*, only one subgroup (*Alpavirinae*) was found to contain temperate members (i.e. detected as prophages). This could be linked to a relatively low number of complete bacterial genomes from aquatic environments. However, the absence of *Microviridae* prophages in *Enterobacteria*, which have been far more thoroughly studied, as well as the absence of detection of any new prophage even with the new *Microviridae* genomes described here, suggests that the use of the lysogenic cycle is likely to be rare among *Microviridae.*

The lysogenic cycle of some of the *Alpavirinae* and the presence of a horizontally transferred peptidase in several of their genomes made it possible to deduce potential host organisms for these phages. As *Alpavirinae* prophages have been found only within *Bacteroidetes* genomes, and the *Alpavirinae* peptidase genes are most similar to genes from *Bacteroidetes* genomes as well, members of the *Alpavirinae* group are likely to infect members of this bacterial phylum. Interestingly, free-living *Alpavirinae* closely-related to *Prevotella* prophages were only found in a lung sample, whereas *Alpavirinae* related to *Bacteroides* prophages were found in different human stool samples. This finding is consistent with the fact that most of the sequenced *Prevotella* strains have been isolated from oral samples, while *Bacteroides* are thought to be primarily associated with gut flora.

The absence of described prophage for *Gokushovirinae* makes it impossible to be conclusive regarding the potential host(s) of these viruses. Yet, 4 peptidases from human gut gokushoviruses form a common clade with genes from a marine bacterium (*Ahrensia* sp. R2A130) and 7 human gut bacteria belonging to genera *Faecalibacterium* and *Anaerofustis* (Fig. 3B, Fig. S9). Both of these genera are members of the order *Clostridiales* (phylum *Firmicutes*), thus it is tempting to speculate that at least some members of the *Gokushovirinae* might infect Gram-positive bacteria.

## Materials and Methods

### Viromes Data Set

A set of 95 viromes available in public databases were downloaded and used in this study (Table S1). Lake Pavin and Lake Bourget viromes were previously described in [45], viromes identified with a number from 12 to 87 in [46], Lake Limnopolar in [6], human lung viromes in [47], human gut and human faeces viromes in [25] and [18], hot springs viromes in [48] and virome JCVI_mv858 is part of the GOS dataset [49]. The 95 viromes span viral communities from the 3 main aquatic ecosystems studied so far (i.e. seawater, freshwater and hypersaline) as well as communities associated with different eukaryotes (fish, coral and mosquito), human lungs and human gut.

### Complete Genome Identification

All viromes were assembled (Table S1), and screened for circular contigs with significant sequence similarity with *Microviridae* genes (tBLASTx, threshold of 50 on bit score). Viromes were assembled using Newbler 2.6 (454 Life Sciences), using the stringent threshold of 98% identity on 35 bp. In addition to the contigs assembly, Newbler software detect putative links between different contigs, usually used to create scaffolds. The contigs linked to themselves (i.e. the end of the contig is similar to the start of it) were thus considered as circular DNA sequences, and searched for *Microviridae*-like genes via tBLASTx (threshold of 50 on bit score). After a first iteration of this search step, all *Microviridae* genomes retrieved were used as query in a second iteration, to detect more distant homologies (i.e. contigs with genes not significantly similar to known *Microviridae*, but significantly similar to contigs retrieved in the first iteration). A fasta file containing the raw sequences of the 81 *Microviridae* genomes assembled in this study, alongside the annotation of each genome in separated genbank-formatted files, in a zip archive (available through Dryad Digital Repository, doi:10.5061/dryad.8ht80; http://dx.doi.org/10.5061/dryad.8ht80).

In addition, complete bacterial genomes from Refseq database and genomes currently assembled from the NCBI were looked for *Microviridae*-like genes via tBLASTx (threshold of 50 on bit score) in order to identify new prophages related to *Microviridae.*

### Annotation of Complete Genomes

An ORF prediction was processed using Glimmer 3.02 [50] for each circular contig identified as a *Microviridae* genome. The predicted ORFs were compared to the sequence database NR using BLASTp and best BLAST hit were conserved. In order to identify and annotate genes not predicted by Glimmer, intergenic regions of the genomes were also compared to NR using BLASTx.

### Analysis of Proteins from the New Circular Genomes

Sequences similar to the Major Capsid Protein (VP1 in *Gokushovirinae,* Protein F in *Microvirus*) were retrieved from known sequenced genomes and complete genomes generated from viromes. The VP1 sequence retrieved as prophage in *Prevotella bergensis* genome was not included in the analysis, since the prophage is split among two scaffolds [17], and thus a genome map is difficult to draw from it. Still, its presence did not modify the tree topology.

A multiple alignment of these VP1 protein sequences was done using Muscle [51]. Mega 5 [52] was used to generate a Neighbor-joining phylogenetic tree from this alignment. A custom-designed Perl script was used to calculate the percentage of identity between each pair of protein sequences, based on the multiple alignments computed with Muscle [51]. Jalview [53] was used to visualize RCR I motif manually on the multiple alignment.

### Structural Modeling and Model Quality Assessment

VP1 homologues from each of the analyzed *Microviridae* subgroup were aligned using PROMALS3D [54] and analyzed for the presence and location of insertions with respect to the sequence of ΦX174 protein F [11]. VP1 sequences of Pavin_279 and BMV5 prophage from *Prevotella bucalis* (GI:282877220; [17]) were chosen as representatives of subgroups *Pichovirinae* and *Alpavirinae*, respectively. Three-dimensional model of the Pavin_279 VP1 was generated using a multi-template (ΦX174 F, PDB ID:1CD3 and SpV4 VP1, PDB ID:1KVP) modeling with MODELLER v9.10 [55] The BMV5 VP1 model was obtained using I-TASSER, which uses a combination of *ab initio* and homology-based approaches for structural modelling [56]. The initial Pavin_279 and BMV5 VP1 models were optimized via multiple rounds of loop refinement with MODELLER v9.10. The stereochemical quality of the models was then assessed with ProSA-web [57]. The final Pavin_279 and BMV5 VP1 models had the quality Z-scores of −6.57 and −6.73, respectively, which were comparable to those of the template structures (-6.4 for ΦX174 F and −6.14 for SpV4 VP1). Comparison and visualization of the structural models was performed with VMD [58] and UCSF Chimera [59].

### Peptidase Phylogenetic Tree

Reference peptidase sequences were taken from the NR database, based on a BLASTp of the *Microviridae* peptidases (threshold of 90 on bit score). Peptidases from *Bacteroides* and *Prevotella* genomes were added to the dataset, as *Microviridae* prophages had been detected in each of these genera. The multiple alignment was computed using Muscle [51], and the maximum-likelihood phylogenetic tree was computed with FastTree [60].

### Major Capsid Protein Detection and Affiliation from Linear Sequences

The unassembled reads from the set of viromes used in this study were screened for sequences homologous to major capsid protein, and these sequences were affiliated via a best BLASTx hit against a database formed of all VP1 from the complete *Microviridae* genomes both published and assembled in this study (threshold of 50 on BLAST bit score).

## Supporting Information

Figure S1 **Boxplot of genome sizes within each clade. Affiliations were based on the major capsid protein phylogenetic tree (** **Fig. 1** **).**
(TIFF)

Figure S2 **Heatmap based on the percentage of identity computed from the major capsid protein multiple alignment.** Scale is indicated on the top left, with the distribution of the percentages of identity. The genome affiliation is indicated above the map, and groups are framed on the heatmap.
(TIFF)

Figure S3 **Multiple amino acid alignment of the major capsid protein.** Large insertions (more than 10 aa) are framed and identified from A to G. The insertion retrieved in all *Microviridae* but *Enterobacteria* phages known to induce mushroom-like structure is identified as the insertion E. One or several sequences were taken for each group, φX174 for *Enterobacteria* phages, CF7ML00001 and *Prevotella Buccalis* for *Alpavirinae,* Pavin_00723 for *Pichovirinae*, *Chlamydia* phage Chp2 and Bourget_00154 for *Gokushovirinae* and *Spiroplasma* phage 4.
(TIFF)

Figure S4 **A boxplot illustrating length variation of the ‘mushroom-like’ protrusion-forming insertions in the major capsid proteins of** ***Gokushovirinae*** **,** ***Alpavirinae*** **, and** ***Pichovirinae*** **.** The insertion lengths are plotted as a function of the *Microviridae* subgroup (A) and ecosystem type (B).
(TIFF)

Figure S5 **Alpaviral VP1 in the context of the entire virion. Pseudoatomic model of the gokushovirus SpV4 virion (PDB ID:1KVP) with one of the capsomers substituted with the structural model of the alpaviral VP1 (** ***Prevotella bucalis*** **prophage BMV5).** The hot-spots in the alpaviral VP1s where specific insertions (>15 aa) with respect to the BMV5 VP1 sequence were detected are indicated with orange spheres. The length of the largest insertion at each of the hot-spots is indicated along with the name of a corresponding viral genome. HF, human feces.
(TIFF)

Figure S6 **Alignment of the conserved motifs of the superfamily I rolling-circle replication protein.**
(TIFF)

Figure S7 **Heatmap based on the percentage of identity from the replication protein multiple alignment.** Scale is indicated on the top left, with the distribution of the percentages of identity. The genome affiliation is indicated above the heatmap, and groups are framed on the heatmap.
(TIFF)

Figure S8 **Heatmap based on the percentage of identity detected on the capsid assembly protein multiple alignment.** Scale is indicated on the top left, with the distribution of the percentages of identity. The genome affiliation is indicated above the heatmap, and groups are framed on the heatmap.
(TIFF)

Figure S9 **Maximum-likelihood phylogenetic tree based on peptidase M15_3 protein sequences.** Each reference sequences is identified by its name, followed by its gene id. *Alpavirinae* sequences are highlighted in pink, *Gokushovirinae* in blue, and viral reference sequences are marked with a black circle.
(TIFF)

Table S1 **List of viromes assembled. For each virome, the number of circular contigs identified as complete** ***Microviridae*** **genome is indicated.** The web-server hosting the datasets are : NCBI (www.ncbi.nlm.nih.gov), MG-Rast (http://metagenomics.anl.gov), and Metavir (http://metavir-meb.univ-bpclermont.fr). When available, the methodology used to purify viral particle is indicated (CsCl : Cesium Chloride, PEG : Polyethylene Glycol, LASL : linker amplified shotgun library and MDA : phi29-mediated multiple displacement amplification). *2 contigs were detected for virome 35 Marine_Sar_Vir, but they corresponded to the 2 contigs already assembled from this virome, described in Tucker et al., 2011, and were thus discarded.
(DOC)

Table S2 **List of circular contigs similar to complete genomes of** ***Microviridae.*** For each major protein, the gi of the best BLAST hit is indicated with the bit score of the corresponding BLAST. All the sequences and corresponding annotations are available through Dryad Digital Repository, doi:10.5061/dryad.8ht80; http://dx.doi.org/10.5061/dryad.8ht80.
(DOC)

Table S3 **List of the** ***Microviridae*** **peptidase genes detected, with their best BLAST hit against NR database.**
(DOC)
