# Supplementary figures and images for: Evolution and Diversity of the Microviridae Viral Family through a Collection of 81 New Complete Genomes Assembled from Virome Reads
Source: PLoS One. 2012 Jul 11;7(7):e40418. doi: 10.1371/journal.pone.0040418 (PMC3394797; doi:10.1371/journal.pone.0040418)

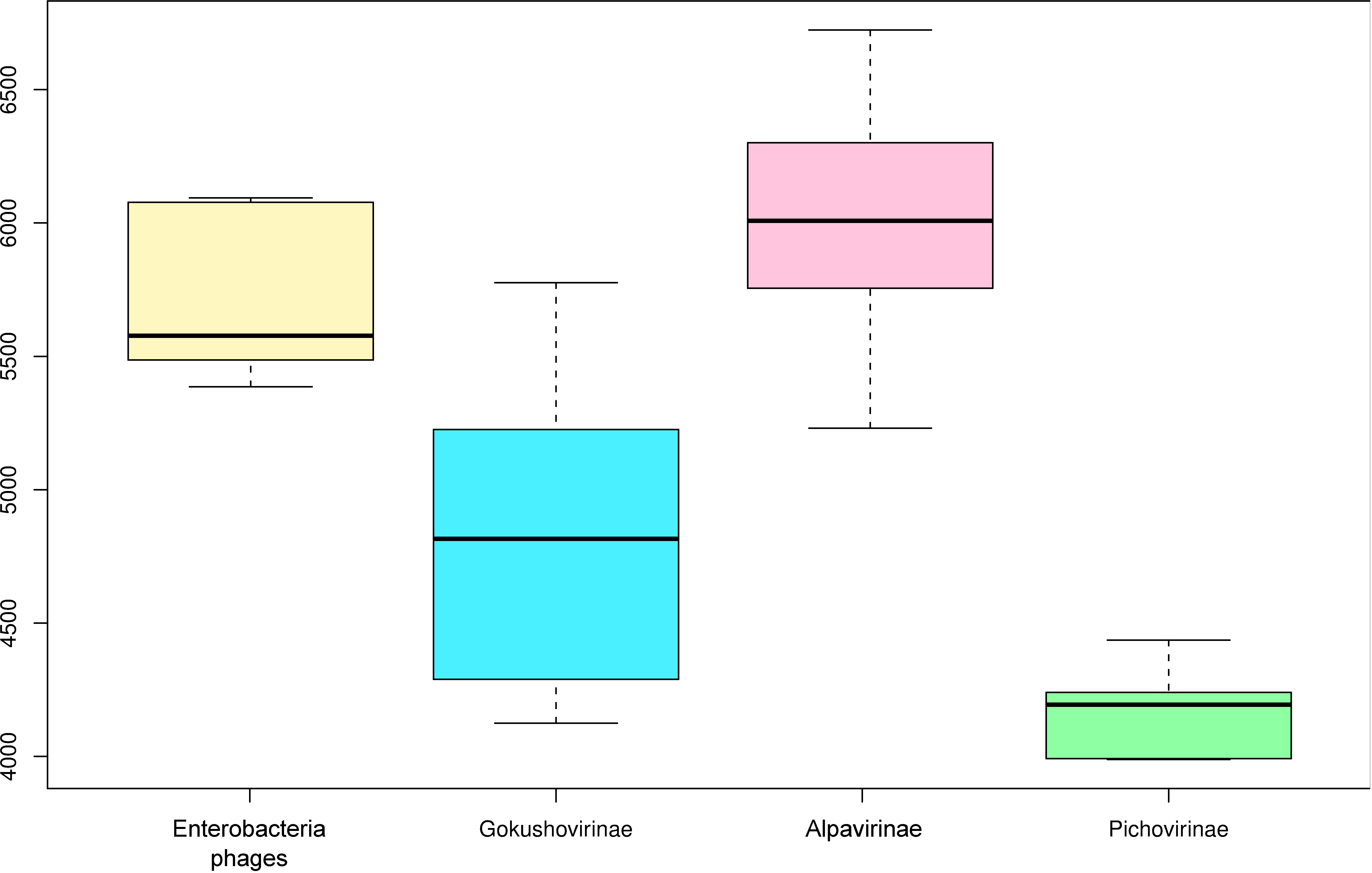

Supplement: Figure S1 — Boxplot of genome sizes within each clade. Affiliations were based on the major capsid protein phylogenetic tree ( Fig. 1 ). (TIFF) [file pone.0040418.s001.tiff]

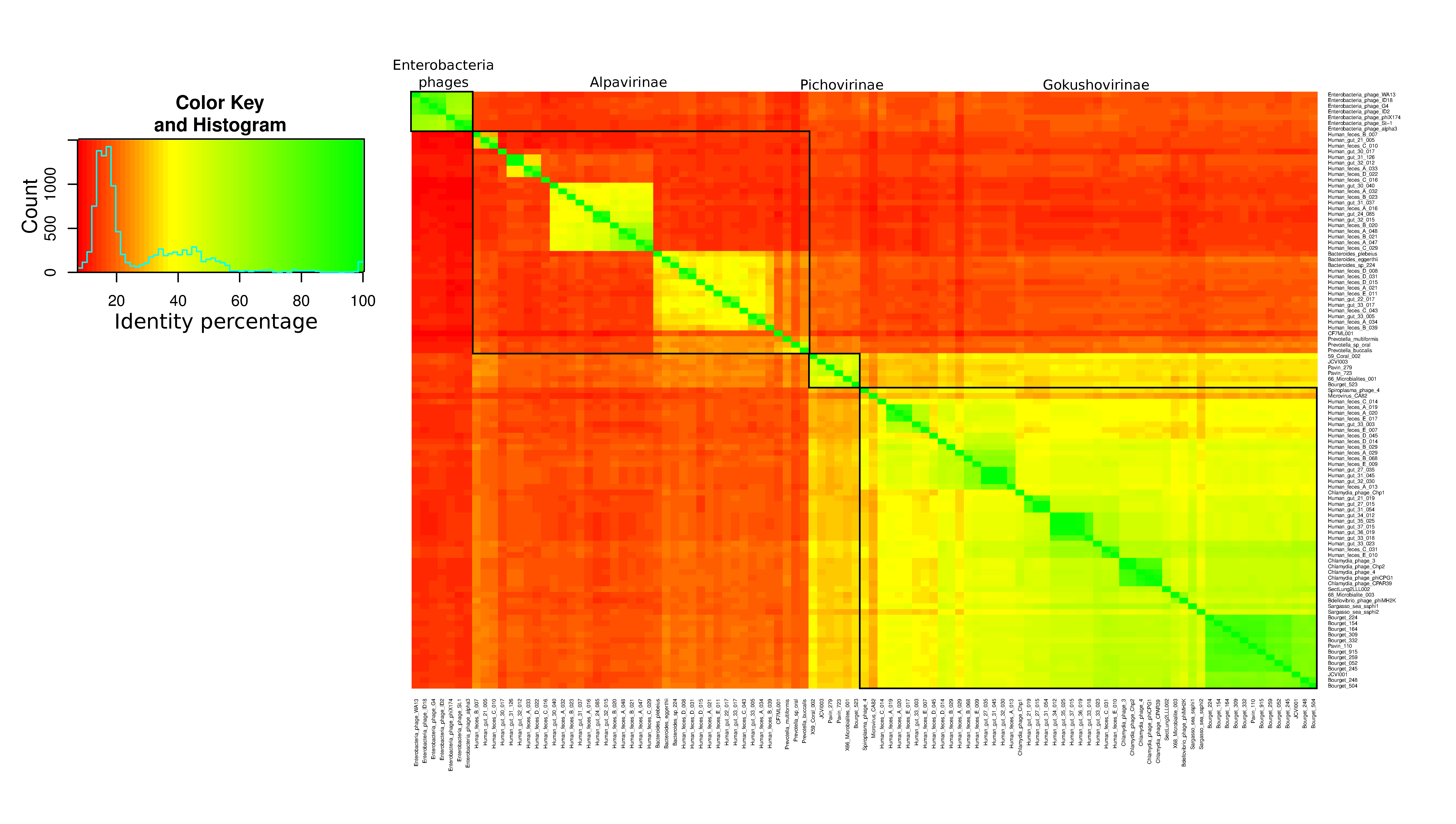

Supplement: Figure S2 — Heatmap based on the percentage of identity computed from the major capsid protein multiple alignment. Scale is indicated on the top left, with the distribution of the percentages of identity. The genome affiliation is indicated above the map, and groups are framed on the heatmap. (TIFF) [file pone.0040418.s002.tiff]

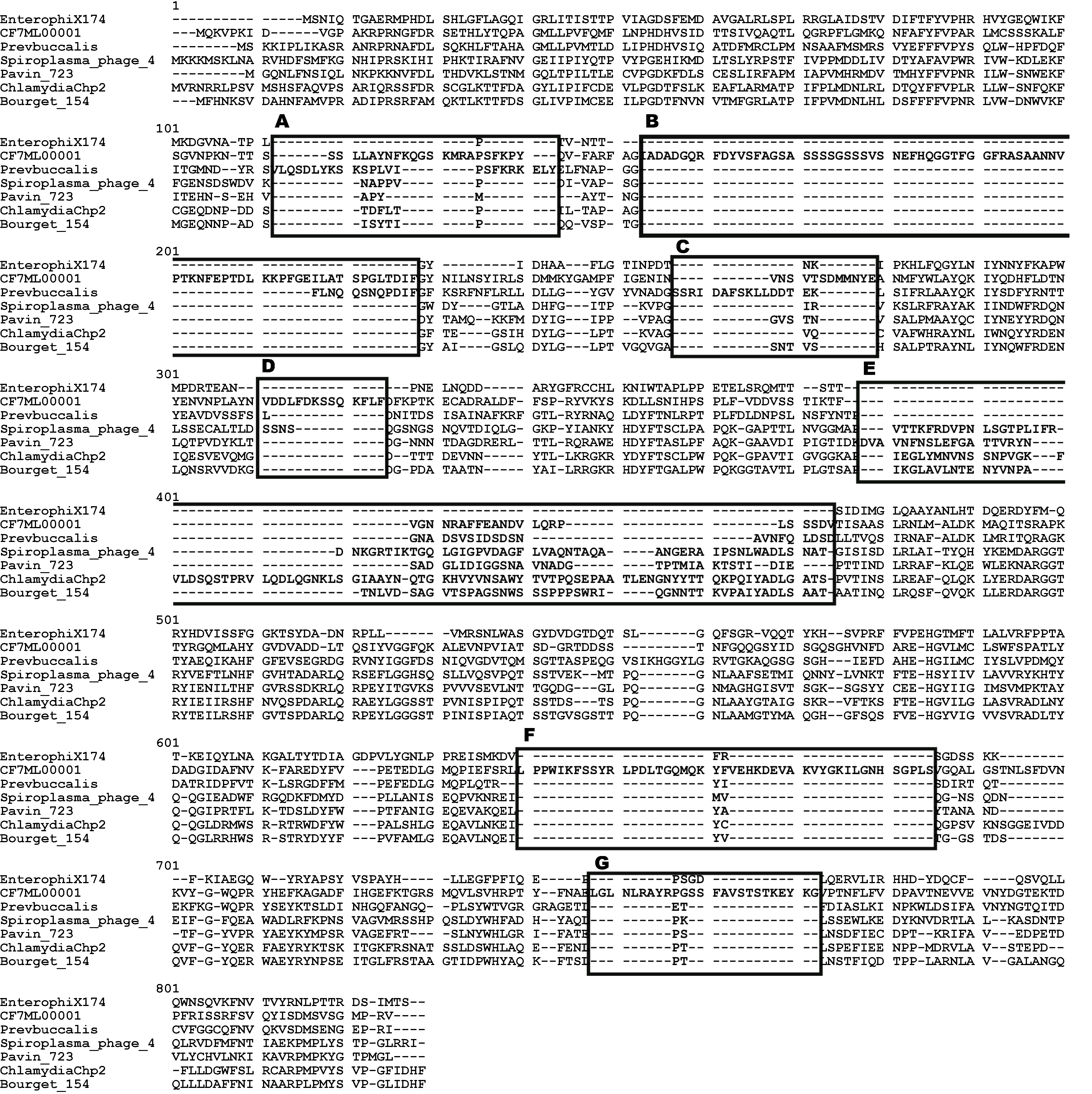

Supplement: Figure S3 — Multiple amino acid alignment of the major capsid protein. Large insertions (more than 10 aa) are framed and identified from A to G. The insertion retrieved in all Microviridae but Enterobacteria phages known to induce mushroom-like structure is identified as the insertion E. One or several sequences were taken for each group, φX174 for Enterobacteria phages, CF7ML00001 and Prevotella Buccalis for Alpavirinae, Pavin_00723 for Pichovirinae, Chlamydia phage Chp2 and Bourget_00154 for Gokushovirinae and Spiroplasma phage 4. (TIFF) [file pone.0040418.s003.tiff]

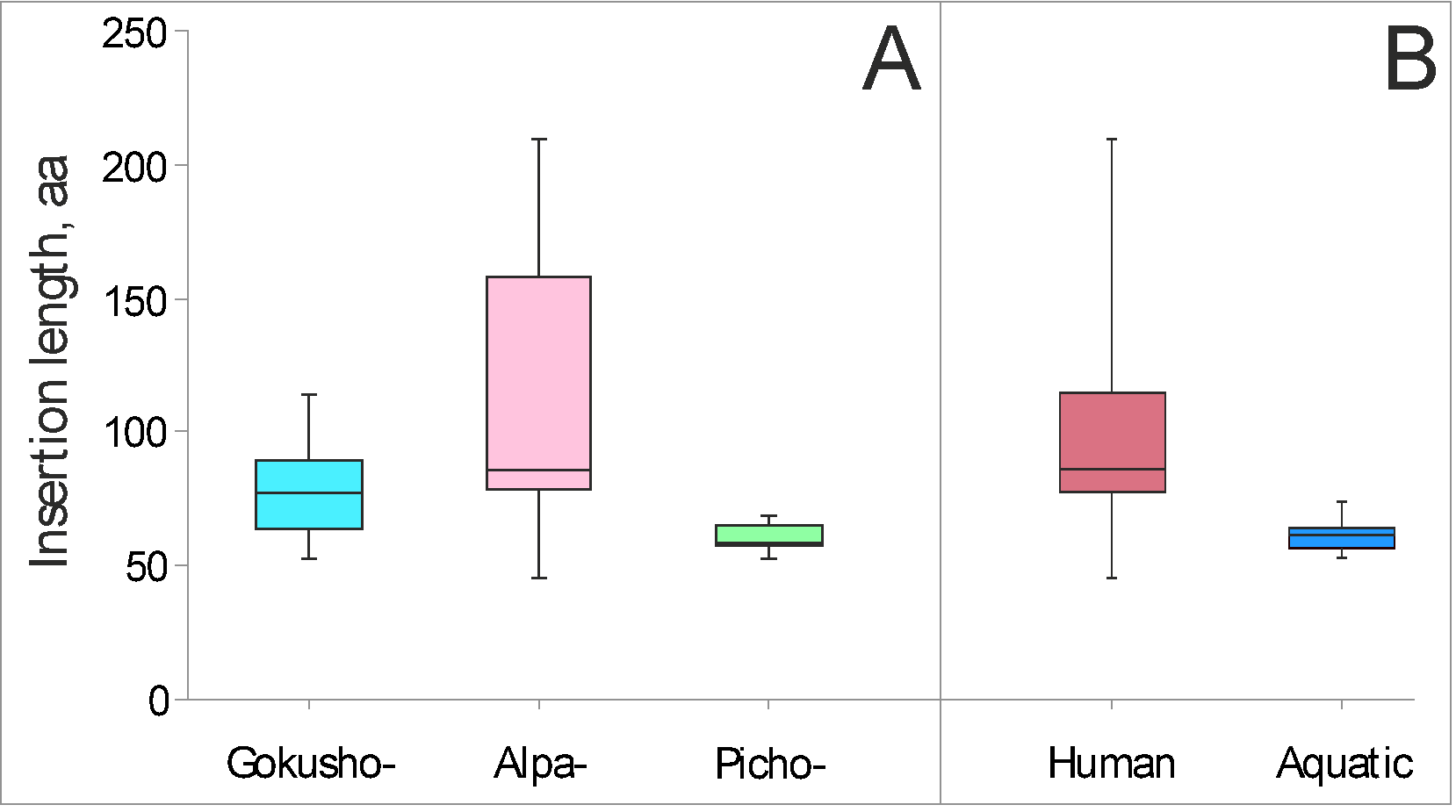

Supplement: Figure S4 — A boxplot illustrating length variation of the ‘mushroom-like’ protrusion-forming insertions in the major capsid proteins of Gokushovirinae , Alpavirinae , and Pichovirinae . The insertion lengths are plotted as a function of the Microviridae subgroup (A) and ecosystem type (B). (TIFF) [file pone.0040418.s004.tif]

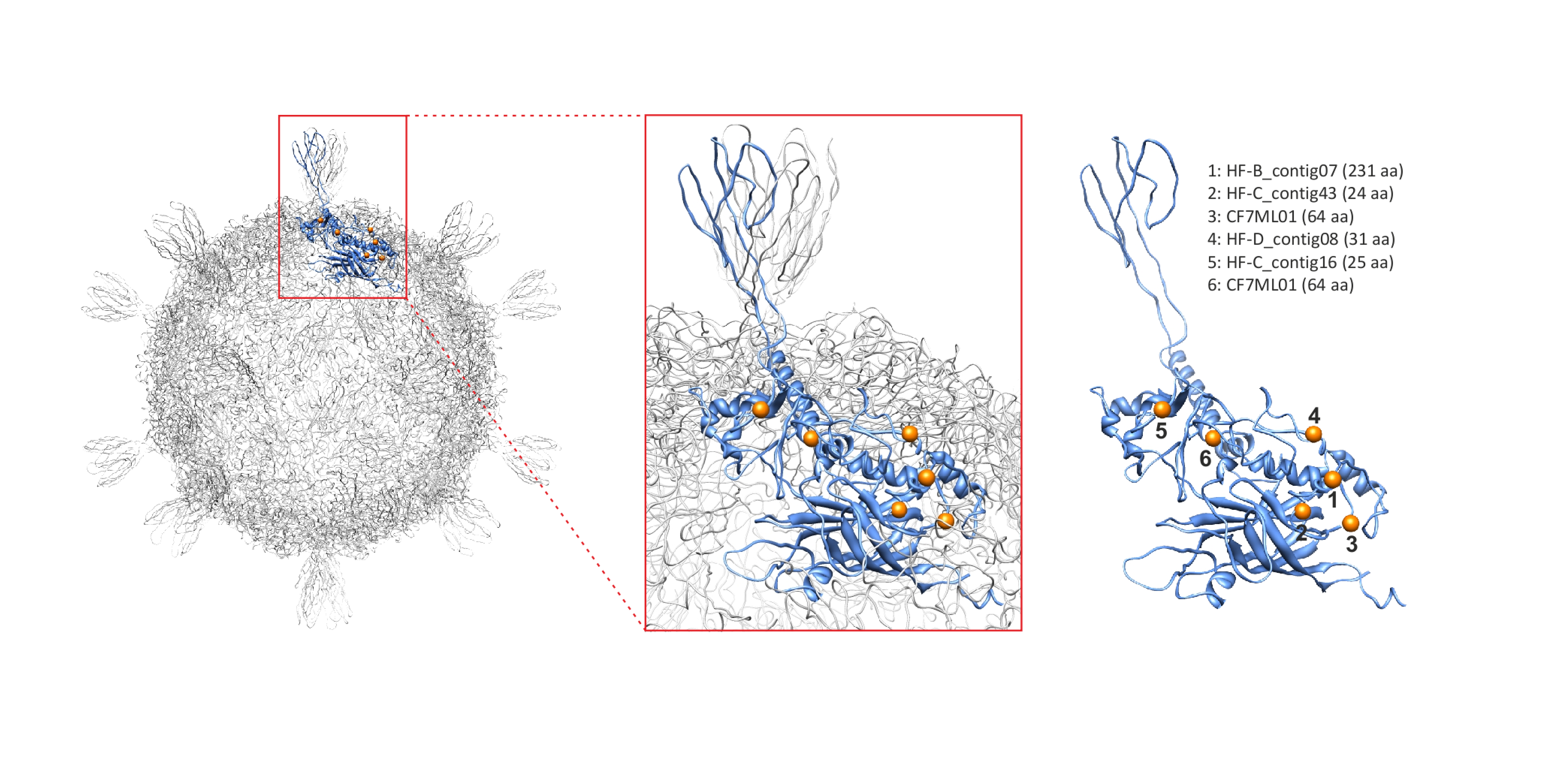

Supplement: Figure S5 — Alpaviral VP1 in the context of the entire virion. Pseudoatomic model of the gokushovirus SpV4 virion (PDB ID:1KVP) with one of the capsomers substituted with the structural model of the alpaviral VP1 ( Prevotella bucalis prophage BMV5). The hot-spots in the alpaviral VP1s where specific insertions (>15 aa) with respect to the BMV5 VP1 sequence were detected are indicated with orange spheres. The length of the largest insertion at each of the hot-spots is indicated along with the name of a corresponding viral genome. HF, human feces. (TIFF) [file pone.0040418.s005.tiff]

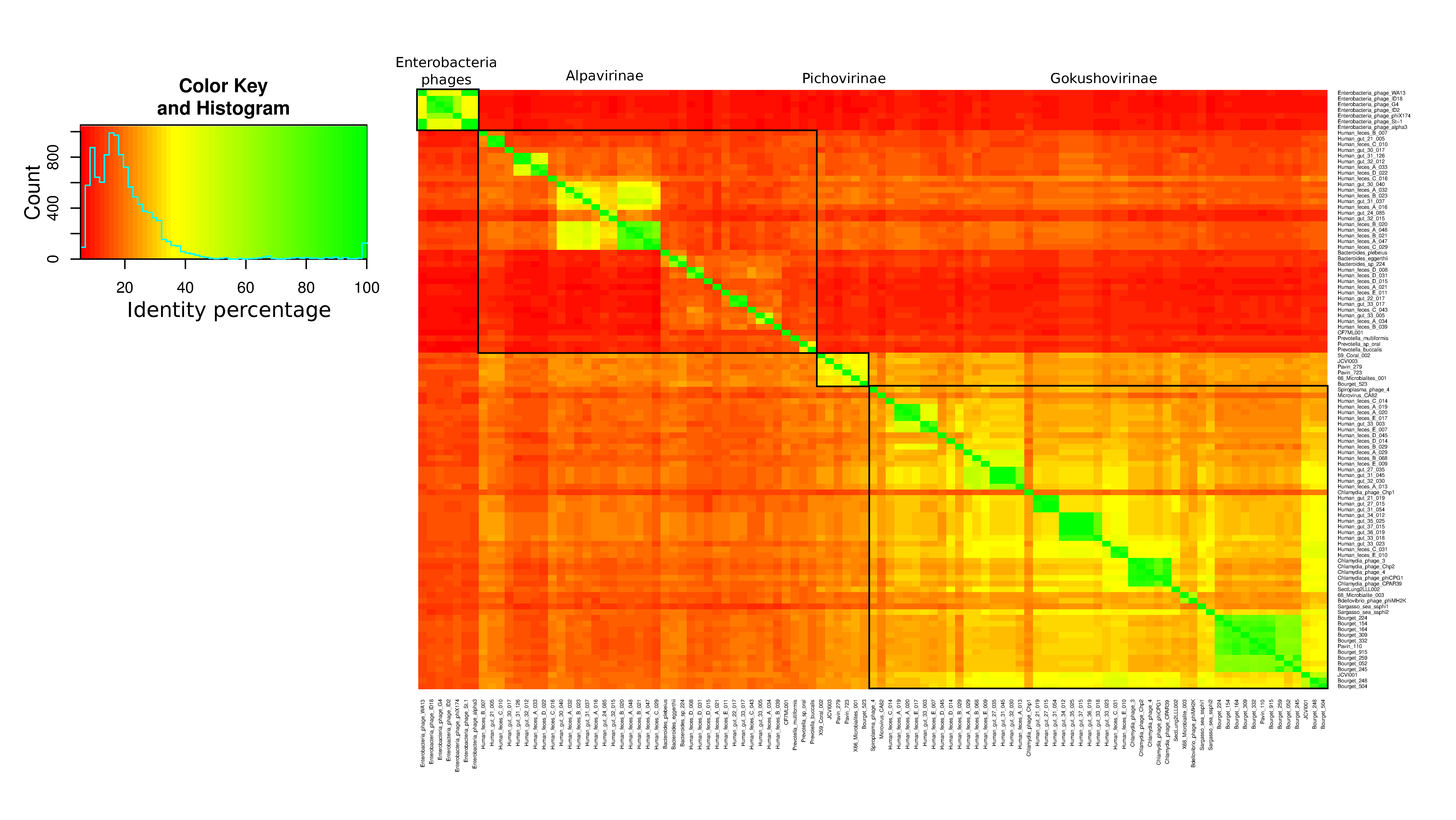

Supplement: Figure S7 — Heatmap based on the percentage of identity from the replication protein multiple alignment. Scale is indicated on the top left, with the distribution of the percentages of identity. The genome affiliation is indicated above the heatmap, and groups are framed on the heatmap. (TIFF) [file pone.0040418.s007.tiff]

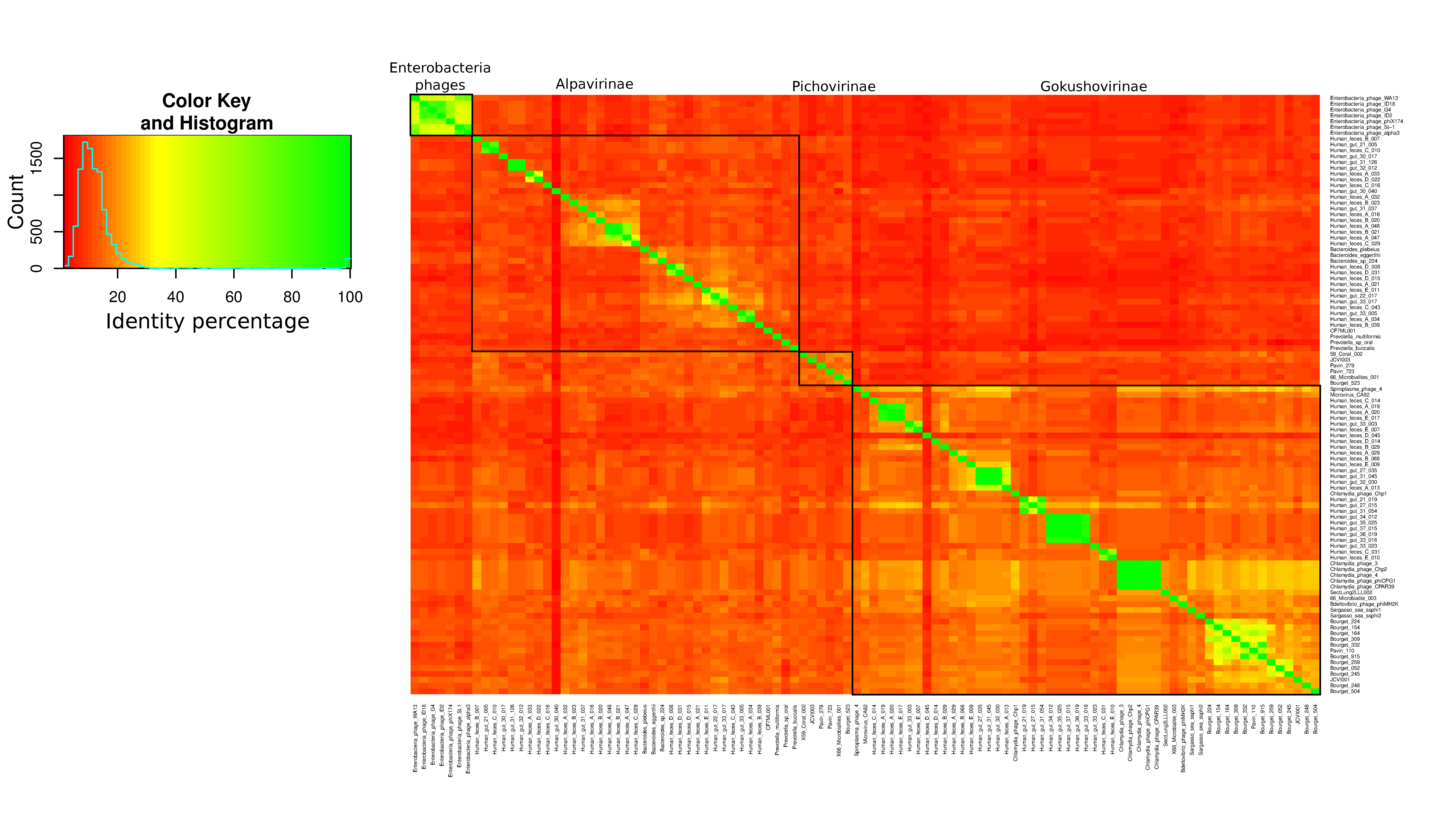

Supplement: Figure S8 — Heatmap based on the percentage of identity detected on the capsid assembly protein multiple alignment. Scale is indicated on the top left, with the distribution of the percentages of identity. The genome affiliation is indicated above the heatmap, and groups are framed on the heatmap. (TIFF) [file pone.0040418.s008.tiff]

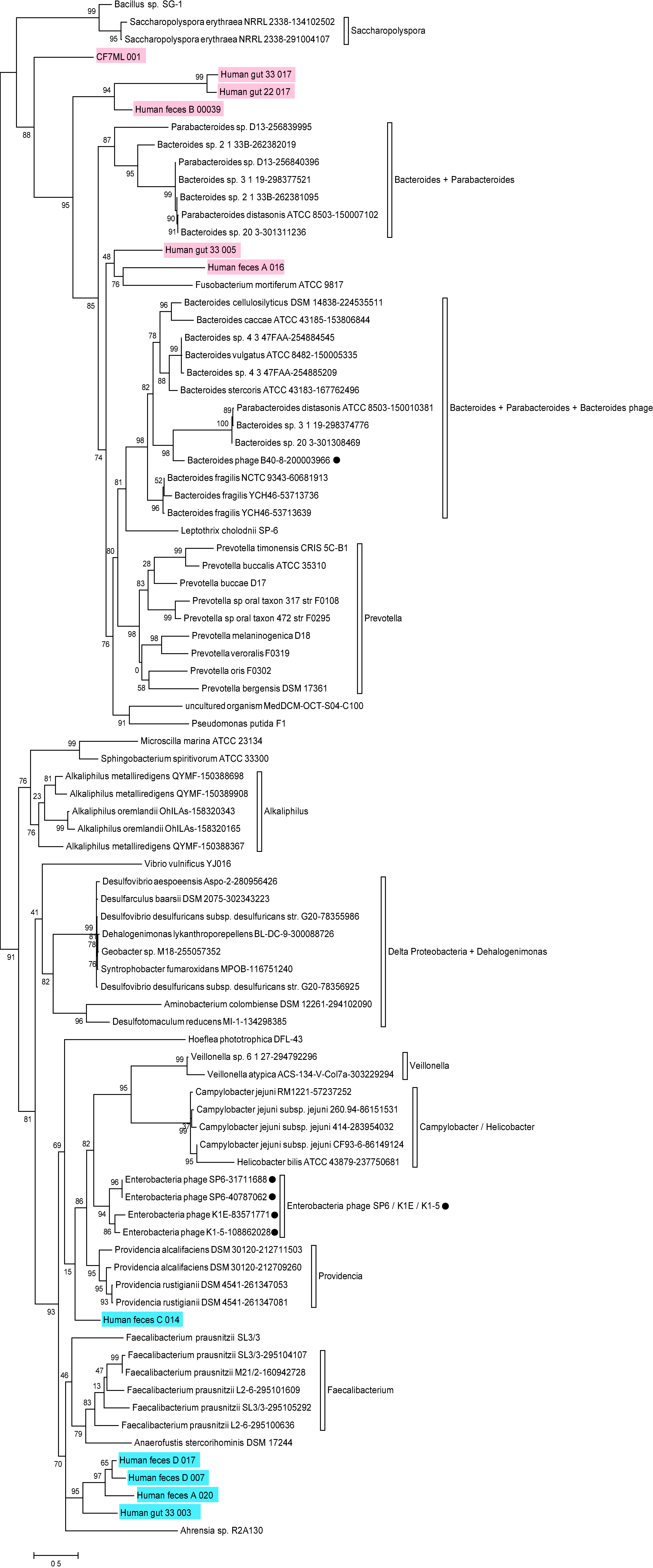

Supplement: Figure S9 — Maximum-likelihood phylogenetic tree based on peptidase M15_3 protein sequences. Each reference sequences is identified by its name, followed by its gene id. Alpavirinae sequences are highlighted in pink, Gokushovirinae in blue, and viral reference sequences are marked with a black circle. (TIFF) [file pone.0040418.s009.tiff]
