# Supplementary material for: Evolution and Diversity of the Microviridae Viral Family through a Collection of 81 New Complete Genomes Assembled from Virome Reads
Source: PLoS One. 2012 Jul 11;7(7):e40418. doi: 10.1371/journal.pone.0040418 (PMC3394797; doi:10.1371/journal.pone.0040418)
Supplement: Table S1 — List of viromes assembled. For each virome, the number of circular contigs identified as complete Microviridae genome is indicated. The web-server hosting the datasets are : NCBI (www.ncbi.nlm.nih.gov), MG-Rast (http://metagenomics.anl.gov), and Metavir (http://metavir-meb.univ-bpclermont.fr). When available, the methodology used to purify viral particle is indicated (CsCl : Cesium Chloride, PEG : Polyethylene Glycol, LASL : linker amplified shotgun library and MDA : phi29-mediated multiple displacement amplification). *2 contigs were detected for virome 35 Marine_Sar_Vir, but they corresponded to the 2 contigs already assembled from this virome, described in Tucker et al., 2011, and were thus discarded. (DOC) [file pone.0040418.s010.doc]

**Table S1 :** List of viromes assembled (CsCl : Cesium Chloride, PEG : Polyethylene Glycol, LASL : linker amplified shotgun library and MDA : phi29-mediated multiple displacement amplification, *2 contigs were detected for virome 35 Marine_Sar_Vir, corresponding to the 2 contigs already assembled from this virome, described in Tucker et al., 2011).
