# Supplementary material for: Evolution and Diversity of the Microviridae Viral Family through a Collection of 81 New Complete Genomes Assembled from Virome Reads
Source: PLoS One. 2012 Jul 11;7(7):e40418. doi: 10.1371/journal.pone.0040418 (PMC3394797; doi:10.1371/journal.pone.0040418)
Supplement: Table S2 — List of circular contigs similar to complete genomes of Microviridae. For each major protein, the gi of the best BLAST hit is indicated with the bit score of the corresponding BLAST. All the sequences and corresponding annotations are available through Dryad Digital Repository, doi:10.5061/dryad.8ht80; http://dx.doi.org/10.5061/dryad.8ht80. (DOC) [file pone.0040418.s011.doc]

**Table S2 :** List of circular contigs similar to complete genomes of *Microviridae.*
