# Supplementary material for: Evolution and Diversity of the Microviridae Viral Family through a Collection of 81 New Complete Genomes Assembled from Virome Reads
Source: PLoS One. 2012 Jul 11;7(7):e40418. doi: 10.1371/journal.pone.0040418 (PMC3394797; doi:10.1371/journal.pone.0040418)
Supplement: Table S3 — List of the Microviridae peptidase genes detected, with their best BLAST hit against NR database. (DOC) [file pone.0040418.s012.doc]

**Table S3.** List of the *Microviridae* peptidase genes detected, with their best BLAST hit against NR database.
